# Medical Electricity

**Published:** 1869-05-01

**Authors:** 


### *Medical Electricity.*

### *The State of our Knowledge concerning the Application of Electricity to the Treatment of Disease.*

REPORTED TO THE ACADEMY OF SCIENCES, PARIS, BY M. BECQUEREL.

**III.—Results obtained by Different Contemporaries.**

After having detailed the electro-physiological phenomena produced by voltaic electricity, which must not be lost sight of in therapeutic applications, we will proceed to speak of results obtained in these applications by the contemporaries, MM. Duchenne (of Boulogne), Namias, Tripier, Poggioli, Scoutetten, Cineselli, Pitet; we have collated the results collected by Remak, long since dead, in order to compare them with those which they have obtained; but in order to utilize them, we will previously revise, in a few words, the facts established hitherto, and of which we have already spoken.

It has been generally recognized by physicians who have preceded those engaged to-day with electro-therapeutics that electrical treatment has for its object to stimulate organs which function imperfectly, and in which life is not extinct, in order to habituate them gradually to function normally. It seemed to result from their observations that the medical employment of electricity is indicated in the three following cases:

1st.—When it concerns the re-establishment of contractility in muscles which have lost it, when the loss of contractility does not involve, or no longer involves, cerebro-spinal lesions.

2d.—When it refers to the establishment of general sensibility, as well as the special sensibility of the organs of sense, this sensibility being abolished or simply diminished.

3d.—When it is necessary to reduce to the normal condition exaggerated or perverted contractility or sensibility.

Have practical physicians obtained other results with new apparatus? It is doubtful.

M. Duchenne (of Boulogne) uses the method of localized electrization indicated by M. Masson, but which he has perfected, generalized and rendered practical. He operates thus:

He takes dry or moist electrodes, by the aid of which he can, at will, concentrate the electrical action upon the skin, or cause it to traverse this latter in order to confine it to organs situated beneath, either in the nerves, the muscles or the bones, and when the epidermis is very thick, the discharge traverses only the dermis and produces sparks, and a peculiar affection, without occasioning any physiological phenomenon.

If he places upon two points of the skin one of the rheophores moist and the other dry, the part upon which this latter is placed experiences a superficial sensation which is cutaneous. In this case, according to M. Ducheune, the recombination of the two electricities is effected in the dry portions of the epidermis after having traversed the dermis by means of the moist rheophore. By moistening, very slightly, the skin, at the points where the epidermis is very thick, there is produced, in the parts to which the dry rheophores are applied, a comparatively superficial sensation, stronger than the preceding, without sparks or crepitation.

If the skin and the rheophores are very moist, there are no longer observed either sparks, crepitation, or burning sensations; but there are manifested very variable phenomena of contractility or of sensibility, according as muscle, nerve, or osseous surface may be involved.

Hence he deduces the consequences that by induced currents electrical power in the skin is arrested at will; that without incision or puncture it may be traversed, and the action of the current may be limited to the organs which it covers, that is to say, to muscles, to nerves, and even to bones. M. Ducheune, in the application of his process, has used, successively, the electricity of machines, of the Leyden-jar, of the voltaic pile, and of the induction apparatus, as most resembling muscular electricity, this last being essentially medical, that is to say, it has been able to induce contraction in each of the muscles, singly or of their sheathes.

The following are the results which he obtained:

1st.—He regards as completely demonstrated, the utility of the electrical treatment applied to consecutive paralysis, traumatic lesions of nerves, and to the paralysis from fatty degeneration of infancy. He asserts that at the beginning of these diseases, the degree of the lesion may be recognized by the aid of the electrical contractility and sensibility of the paralyzed muscles.

2d.—Electricity is equally applicable, but with less certainty, to the paralyses termed spinal, and to those essentially rheumatismal or hysterical, according as they are more or less localized or generalized; but as these affections may be cured spontaneously, or disappear temporarily, the real value of the electrical treatment can not be determined.

3d.—Neuralgias, generally, with the exception of facial neuralgias, are cured by electro-cutaneous excitation.

4th.—Rheumatic muscular pains are rapidly cured by electrical treatment.

5th.—Cutaneous or muscular hyperæsthesia, and cutaneous anæsthesia of hysterical or saturnine origin are advantageously modified by electro-cutaneous excitation.

6th.—He has treated successfully, according to his own statement, local affections, such as paralysis of the seventh, of the third and of the sixth pairs, aphonia, deafness, paralysis of the bladder, and some cases of intestinal strangulation.

7th.—The application of electricity to chorea, to scrivener's palsy, of gout, and of amorosis, has produced results nearly negative. M. Mamias uses, ordinarily, a circular pile of cups formed of two hundred elements charged with salt water. The force of this pile diminishing rapidly, he replaced it by another, and this again by a third, in order to give time to the couples to be depolarized; this was the infancy of these piles. He is positive of having determined that by their use, the calorific or other effects which are inevitable with the cutaneous current piles used to-day, are avoided. The results obtained by him are the following:

1st.—Intermittent currents leave no durable impression upon the living body. Moderate shocks keep the nerves and muscles in exercise, and do not oppose vital reaction. Afflux of blood and increased nutrition follow repeated shocks.

2d.—If the shocks are excessively strong, but yet not sufficient to kill the animal, they leave no disease.

3d.—Continuous currents, too, prolonged produce disease.

4th.—He has recognized the influence of direction upon human nerves, which had been considered null.

5th.—He has determined the cases of paralysis in which the cure is complete, and those in which there is only amelioration, with intermittent currents which are preferable to others; he employs centrifugal currents in paralysis of motion, and centripetal in paralysis of sensation.

6th.—In neuralgias or neurosis there is no fixed rule; sometimes it is necessary to employ continuous, and sometimes intermittent currents in one sense or another.

7th.—In affections of the vascular and lymphatic system continued currents are necessary, as opposed to affections of the nervous and muscular system, which need intermittent currents.

8th.—He has demonstrated, according to his own account, that the employment of electro-muscular contractility in order to find the seat and the nature of paralysis is an error. M. Poggioli uses exclusively static electricity in the treatment of disease as it was administered before the discovery of the pile, basing his opinions upon the theory of Franklin. He especially recommends electrified water for imbibition, and electric baths.

M. Tripier has presented a *Treatise on Electro-Therapeutics* in which he passes in review all the methods employed, and the results attained, which he seeks to explain by means of theories. He considers as original:

1st.—His reflections upon the action of induction currents according to their direction and intensity;

2d.—The use of excitors of different kinds, especially of charcoal;

3d.—The surgical indications of the galvano-caustic chemical method which he has applied to different pathological conditions;

4th.—The explanation of anæsthesia;

5th.—Experiments upon gustatory sensations induced by mediate or immediate galvanism of the tongue;

6th.—The cure of a certain number of diseases;

7th.—The treatment of hyperplasias of the connective tissues of contractile organs, especially of the uterfous and the prostate, etc.

M. Scoutetten presented to the concours a work having for its title *Electricity considered as the principal cause of the action of mineral waters upon the organism*, and in which he treats, from his stand-point:

1st.—Electrical action of mineral waters upon the exterior and the interior of the human body, whether taken in the form of bath or of drink.

2d.—Electricity of the blood of man and of living animals, and of the electrization of transported mineral waters.

In addition to this work M. Scoutetten presented special memoirs in which he has developed the different questions, out of which he has formed a body of doctrine.

M. Ciniselli presented a little treatise in which is set forth a synopsis of his investigations into chemical galvano-cautery, a method pointed out thirty years ago by one of your committee, and which he, also, in connection with M. Breschet, applied at l'Hotel Dieu, of Paris.

Chemical, is distinguished from thermal galvano-cautery in this regard, that the latter cauterizes by means of the heat produced in a metal wire traversed by an electrical current of a certain intensity, whilst the other effects cauterization by the aid of an acid or an alkali, separated from a solution by the chemical action of the current. He employs for this object either a simple circuit, or a circuit in which there is a pile. According to the direction of the current, he directs upon the diseased part an acid or alkaline caustic in the nascent state, and, consequently endowed with great energy. M. Ciniselli enumerates in his treatise the cases in which he effected cures by operating upon tumors of different kinds, in different pathological conditions. M. Nelaton, by the aid of similar methods, has removed nasopharyngean tumors. No one can fail to felicitate M. Ciniselli upon his efforts to apply electro-chemistry to therapeutics; and your committee, likewise, is pledged to persevere in the same direction.

Doctor Pitet has devoted himself to the establishment of a parallel between the physiological and pathological effects produced by interrupted and by continuous currents, and, to demonstrate the superiority of the therapeutic action due to the most feeble over that of the most energetic induced currents. He has, moreover, arrived at this conclusion: that the best mode of application is that of continuous currents. The results of his investigations are succinctly subjoined:

Induced and continuous currents produce effects essentially different; the first tend to produce constantly a condition inverse to that which exists at the moment of their application; that is to say, their initial specific effect being constantly the same as the pathological condition which they destroy, from which it results that their therapeutic effect is inverse of the first.

Continuous currents, on the contrary, produce upon the affected organ the same effects which they induce in the physiological state, that is to say, a relaxation, a dilatation, etc.

According to his observations, energetic induced currents, applied in the physiological, as well as in the pathological condition, fatigue the subjects, and often aggravate the morbid state; they alter and destroy sensory and motor irritability, whilst continuous currents, on the contrary, are easily tolerated by the organism; they are employed advantageously in vascular congestions; their influence is such, that it should receive serious consideration in therapeutics.

M. Pitet relates a certain number of facts which he considers as demonstrating the principles just indicated.

No one can fail to approve the course of the author in studying, successively, the physiological action of electricity upon an organ in the normal state, and upon the same organ in a morbid condition. This is the true course to be pursued in order to arrive at a correct appreciation of the real therapeutic action of electricity.

M. Remak used piles with constant currents, and piles which have not this property. The results of these experiments are subjoined:

1st.—The continuous current, to a supportable degree, acts upon central organs, and induces, by reflex movements, contractions, even in antagonistic groups of muscles.

2d.—The continuous current increases, within certain limits, the excitability of the nerve, instead of weakening it, and that, too, in the sensitive, as well as in the motor nerves.

3d.—He has affected the resolution of paralytic contractions by means of continuous currents. It is this process which, under favorable circumstances, heals paralysis for the treatment of which intermittent currents are prejudicial.

4th.—He has, likewise, cured old paralysis.

5th.—He has experimented upon patients affected with rheumatic contractions or pains; having transmitted, during about five minutes, a current of fifteen or twenty elements with sulphate of copper, through the muscles of the shoulder, the patient raised his arm, and placed it upon his head.

6th.—He next sought, without success, to discover if the continuous current of a certain force was not of a character to produce some disorder in the organism. The employment of interrupted currents only aided him in particular cases, and which are not even very frequent. If the results just indicated be compared it will be perceived that physicians are not in accord, either in the mode of treatment, or in the results attained.

Indeed, M. Duchenne employs, with success, intermittent currents in the majority of cases, treatment which M. Remak rejects as hurtful, giving the preference to continuous currents. M. Namias pretends to demonstrate that the electric diagnostic of M. Duchenne for the recognition of the seat of paralysis is false. This last does not recognize in man the hypoanæsthetic (?) properties of continuous currents.

M. Remak, and to some extent M. Pitet, asserts that the continuous current increases within certain limits the excitability of the nerve in place of weakening it. It is this property which has suggested its employment in the treatment of paralysis, in preference to the induced current. It must be added that M. Hifflesheim considered the intermittent current as an excitant and the continuous as a sedative. It should be observed that the hyposthenic action of continuous currents appeared to be very generally recognized, and that physiologists admit that with feeble currents directed successively in inverse manner, there is but very slight hyposthenic action, whilst, when the currents are very intense, this action becomes predominant.

These divergences, as well as others which might be cited, in the results obtained and in the opinions expressed upon the value of this or that proceeding demonstrate the impossibility of deciding, as yet, upon the true therapeutic properties of electricity, whether continuous or intermittent currents are employed, especially when the treatment has not been followed up.

One of two things is certain; either electricity acts effectively, or its action is negative. Upon the first hypothesis it must be concluded that physicians have not experimented under the same conditions of age, of constitution, of vital force, of equal degree of disease, and with electrical appliances having the same intensity; for if every thing had been similar on every hand, there would have been no reasons for not obtaining the same resnlts. Upon the second hypothesis, it would be necessary to admit that nature has done every thing. We are borne to the conclusion, invariably, that the applications have not been made under the same conditions, for it could not be denied that electricity would have acted efficaciously in certain paralyses and other pathological conditions; numerous examples, already old are at hand to sustain it.

### IV.—Observations and Conclusions.

We ask of the Academy permission to present to it some observations, which will not be without utility for therapeutic applications.

Continuous currents and interrupted currents have each their mode of action; the former, by the aid of moistened electrodes, penetrate under the skin into the organs, and produce there effects physical, chemical, calorific, and, perhaps, of transportation, effects dependant upon the intensity of the current and of the conducting power of the parts which they traverse. These parts are, muscles, nerves, their organic elements, vessels, all tissues, etc., between which, the current divides itself in proportion to the conducting power of parties which do not form a homogeneous entity as a metallic conductor; there are branchings, anastomoses, contacts more or less immediate, from which result resistances, light shocks from changes of conductors, which may be only slight shiverings; special actions upon nerves and upon the muscles, of which we have already spoken; effects of heat produced by obstacles to its passage; perhaps chemical effects upon changes of conductors.

Have all these effects in the course of the electro-physiological investigations upon animals, effects which are interesting to know, been analyzed?

The effects of heat may be studied with great precision by the aid of thermo-electric needles; neither chemical effects nor effects of transportation have, as yet, been determined. Moreover, is it not well known, that threads of metal, a bad conductor, such as platinum, are traversed by intense currents?

Who can say that similar effects are not manifested in nerve-fibres, muscle-fibres, capillary-vessels, etc.

All these effects may exercise an influence upon organic functions; upon this point investigation is necessary. It is necessary, moreover, according to the example of M. Namias, in his electro-physiological experiments upon animals, to observe, after their death, what have been the effects produced upon organs, according as continuous or intermittent currents of given intensity have been employed, in order to make their application to man.

Intermittent currents, independently of the physiological effects already mentioned, produce, likewise, heat, during the successive discharges, as has been demonstrated by discharging a Leyden-jar through a metallic wire, and effects of distension as is seen by passing the charge of a Leyden-jar into a thin tube of glass of small diameter, which flies into fragments; these questions must be examined when it is intended to treat the subject scientifically, whilst investigating the therapeutic effects of electricity; it is thus seen how complex is the action of electricity upon organs.

When the general considerations which precede the memoirs and works presented to the commission are reviewed; it is easy to be convinced that the experimenters formed no just idea of the mode of disengagement of electricity in the apparatus which they used. These apparata comprise ordinary electrical machines, voltaic piles with constant currents, electro-magnetic and magneto-electric machines, whose forms and arrangements are very varied. The effects resulting from electricity disengaged during chemical actions is no longer sufficiently considered.

Electricity, whatever may be the source which disengages it, is always of the same nature; it differs from one source to another only by its tension, its quantity, and the duration of its passage. In the pile the tension of the electricity is, in general, weak at the two poles, but it produces energetic physical effects, by reason of the quantity of electricity which passes in the circuit when it is closed.

On the other hand, at the instant of closing the circuit of the pile with a metallic wire, the electric current which traverses this wire produces in it another, by induction, (extra current) proceeding in an inverse manner; this current, whose duration is very short, and may, perhaps, be considered almost instantaneous, tends to diminish, even at the instant of its closure, the intensity of the induction current; upon opening the circuit, there is generated another induced current, directed in the manner of the induction current, which has the same character as the discharges from the Leyden-jar.

Induced currents generated by voltaic currents, or in wires kept apart, differ amongst themselves in intensity, according to the force of the pile, and the length of the wires. They have a peculiar character, whilst in the discharges there are two instantaneous currents directed in an inverse manner, and acting as alternate currents.

Electro-magnetic or magneto-electric apparatus can be constructed only with the object to facilitate the application of electricity by intermittent currents; the effects which they produce differ between themselves only by the intensity of the discharges; it is even possible to obtain similar effects with Leyden-jars which are discharged and recharged more or less rapidly. There are, therefore, special effects relative to the apparatus determined only by the circumstances of the intensity of the duration and of the succession of the discharges.

In general, sufficient importance is not attached to the physiological effects which may be produced by electricity disengaged by the contact of liquids in organized bodies. When two different liquids, conductors of electricity, are in contact, they place themselves always in two different electrical states, whether there should be a chemical reaction between the two, or only a simple admixture; the one acting as the acid liberating positive, and the other egative electricity. These two electricities remain in the static condition so long as the liquids do not form a closed circuit by means of a solid conducting body not permeable. In the static state, the tension of the electricity is so feeble that very sensitive instruments are necessary for its detection. There is, moreover, a recombination of the two electricities in proportion as they become free, even upon the contiguous surfaces, so long as the chemical action or he mixture continues; it is then impossible to perceive how this electricity could exercise any action upon the internal organs, especially from the administration of mineral waters. If these waters are alkaline, in reacting upon the acid secretion which covers the skin, they assume negative, and the secretion positive electricity; the recompositon of the two electricities is effected upon the skin, and the internal organs can experience no effect therefrom.

In the second case, when the circuit is closed by means of a metal, electro-chemical effects are, doubtless, produced; but are there, in the organs of men or animals, conductors suitable for forming closed circuits? What are the solid parts, conductors and non-permeable, which could determine the circulation of electricity disengaged by the contact of liquids during their mixture or when they may react chemically upon each other? None such are known; for there are only the tissues which separate the liquids, and by the intermediation of which the reactions are effected; deprived of these liquids they are not conductors.

It is not sufficient to base a physiological theory upon a fundamental fact. It is necessary to begin by demonstrating this fact.

For the present, the existence of electrical currents in the organs of living man, currents due solely to the reaction of liquids, independently of the employment of metallic conductors, is negatively demonstrated.

